# Tolerating CD47

**DOI:** 10.1002/ctm2.1584

**Published:** 2024-02-16

**Authors:** Jeffrey S. Isenberg, Enrique Montero

**Affiliations:** ^1^ Department of Diabetes Complications & Metabolism Arthur Riggs Diabetes & Metabolism Research Institute City of Hope National Medical Center Duarte California USA; ^2^ Department of Molecular & Cellular Endocrinology Arthur Riggs Diabetes & Metabolism Research Institute City of Hope National Medical Center Duarte California USA

**Keywords:** autoimmunity, cancer, CD47, checkpoint inhibitor, immunotherapy, SIRPα, TSP1

## Abstract

Cluster of differentiation 47 (CD47) occupies the outer membrane of human cells, where it binds to soluble and cell surface receptors on the same and other cells, sculpting their topography and resulting in a pleiotropic receptor‐multiligand interaction network. It is a focus of drug development to temper and accentuate CD47‐driven immune cell liaisons, although consideration of on‐target CD47 effects remain neglected. And yet, a late clinical trial of a CD47‐blocking antibody was discontinued, existent trials were restrained, and development of CD47‐targeting agents halted by some pharmaceutical companies. At this point, if CD47 can be exploited for clinical advantage remains to be determined. Herein an airing is made of the seemingly conflicting actions of CD47 that reflect its position as a junction connecting receptors and signalling pathways that impact numerous human cell types. Prospects of CD47 boosting and blocking are considered along with potential therapeutic implications for autoimmune diseases and cancer.

## Graphical abstract

Connecting the dots on CD47

1. CD47 interacts with ligands along with known and to‐be‐determined (TBD) receptors in *cis* and *trans*.

2. CD47 is the center piece of a pleiotropic receptor‐multiligand interaction network.

3. CD47‐targeted interventions may disrupt homeostatic connectivity and have on‐target effects.

4. CD47 immunomodulation to improve health requires connecting the missing dots.

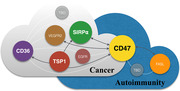

## 1 INTRODUCTION

**FIGURE 1:**
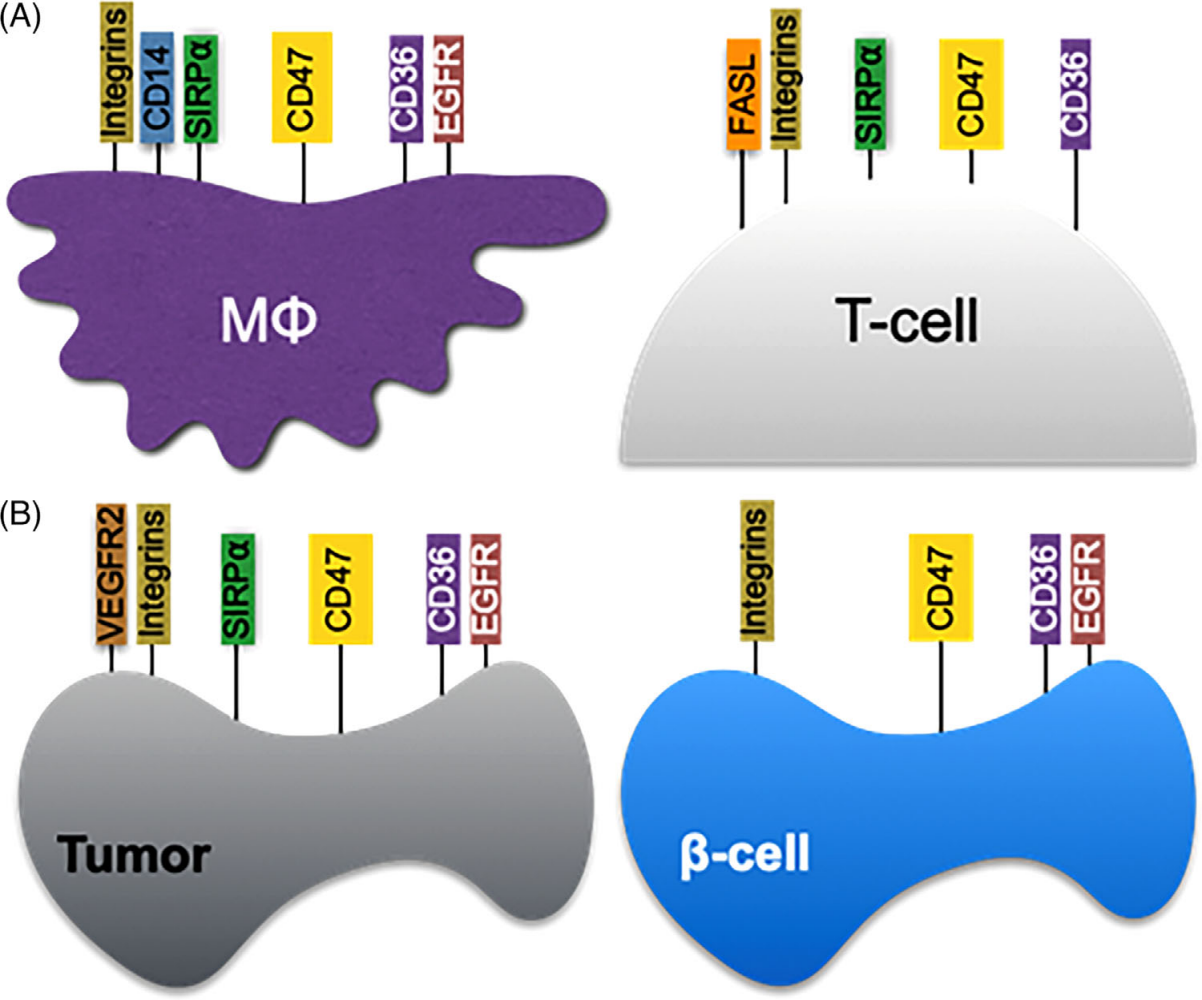
CD47 is omnipresent in normal and tumour cell types, associating with other surface molecules displaying diverse functions, including activation, suppression and cell death. CD47 *cis* activities may contribute to stabilising the expression of cell receptors in the cell synapse (A), eventually leading to synergising or inhibiting the activity of the different associated receptors in multiple cell types. CD47 molecule expression can increase in cancer (B) and in physiological conditions, but a detailed expression kinetics is poorly defined. The association of CD47 in *cis* to the depicted molecules has been consistently determined, although EGFR is yet presumptive. VEGFR2, vascular endothelial growth factor receptor 2; EGFR, epidermal growth factor receptor; FAS (human), aka Fas cell surface death receptor, aka tumour necrosis factor receptor superfamily member 6 receptor.

CD47 is present on the surface of all human and mammalian cells examined. The soluble stress‐reaction protein thrombospondin‐1 (TSP1) is its high‐affinity ligand. Plus it is a *cis* and *trans* interacting partner with other cell surface receptors including vascular endothelial growth factor receptor 2,^1^ FAS, the receptor for FAS ligand,^2^ beta integrins,^3^, ^4^ signal regulatory protein alpha (SIRPα),^5^ CD14,^6^ CD36^7^ and perhaps epidermal growth factor receptor (EGFR)^8^ (Figure 1A). The interaction with CD36 was assumed based on studies using the TSP1‐derived 4N1K peptide but needs to be fully demonstrated, in part, because 4N1K showed CD47‐independent activity.^9^ Adding complexity, CD47 congregates at distinct areas on the membrane of immune^10^ and nonimmune cells^11^ and such aggregations control cell signalling and phagocytosis (Figure 1B).^12^ *Trans* binding with SIRPα promotes clustering and CD47‐targeting antibodies antagonise this.^11^ This is relevant given recent efforts to overexpress CD47 on cells^13^ as a shield against immune cell attack. Nonetheless, interacting with other same‐cell surface receptors, CD47 significantly modified the signalling of each. This emphasises that CD47 connects multiple receptors and signalling pathways. As a consequence, approaches to therapeutically engage CD47 may distort these relationships. Much effort and resources are directed towards making drugs that block CD47.^14^, ^15^ Several CD47 blocking antibodies are in clinical trials to harness the immune system and treat cancer. But given that CD47 is displayed by all primary human cells, unexpected results may come about. The manufacturer of the leading clinical CD47 antibody halted a late Phase 3 trial while companies, including Arch Oncology, AbbVie and ALX Oncology, partially or completely curtailed work on CD47 therapies. A closer look at CD47 identifies invaluable functions beyond serving as an immune checkpoint.

### 1.1 CD47 perturbs cell survival and metabolism

CD47 biding promoted death of anti‐CD3‐activated human T cells.^16^ This was driven by TSP1, necessitated CD47, and involved downstream Gi protein coupled receptor suppression of cyclic adenosine monophosphate (cAMP).^17^, ^18^ The ability of CD47 to stimulate cell death was not limited to immune cells.^19^, ^20^ Despite that, in certain cancers, TSP1‐CD47 stimulated growth.^21^ CD47, on activation by TSP1, also degraded mitochondrial function^22^ and increased production of pathologic reactive oxygen species (ROS) in T cells^23^ and nonimmune cells.^24^, ^25^, ^26^ In the presence of research‐grade CD47 antibodies CC2C6, B6H12, and 2D3, human polymorphonuclear neutrophils effectively trogocytosed T cells while producing excess ROS.^27^ It is odd that 2D3 enhanced cell membrane ingestion as it does not impinge on CD47‐SIRPα mediated engulfment^28^ but this result may indicate effects on CD47‐integrin signalling. In these assays, exogenous soluble SIRPα protein also promoted T‐cell killing^28^ imputing so‐called reverse signalling with SIRPα acting as a ligand for CD47. Further, crystallisation of B6H12 bound to the CD47 ectodomain was achieved,^29^ something helpful as B6H12 instigated the development of a leading clinical CD47 blocking antibody.^30^ Macrophages exposed to a CD47 blocking antibody (IgG1 clone OX101) or soluble CD47 protein produced excessive ROS^31^ via loss of SIRPα inhibition of the NADPH oxidase (Nox) gp91(phox) subunit.^32^ Interestingly, in human arterial vascular smooth muscle cells, TSP1 activation of CD47 targeted the p47phox subunit of Nox to increase superoxide production.^24^ Additionally, TSP1 engaged and phosphorylated nonphagocytic SIRPα to increase ROS.^33^ Further supporting a redox function, activated CD47 inhibited production of vascular endothelial nitric oxide and directly curtailed its canonical downstream signalling through cyclic guanosine monophosphate and respective nucleotide‐stimulated kinases,^34^, ^35^, ^36^, ^37^, ^38^ all indispensable mediators of angiogenesis, tissue perfusion, blood flow and cardiovascular health.

Surface CD47 was decreased on aged red blood cells, suggesting that CD47 leads to senescence.^39^ More compelling, TSP1 activation of CD47 pushed primary human cells from a proliferative to a senescent state,^40^, ^41^ in line with its constraint of requisite self‐renewal transcription factors Oct3/4, Sox2, Klf4 and cMyc.^42^, ^43^, ^44^ This positions CD47 as an accelerant of cellular and organismal aging.^45^, ^46^, ^47^ In cancer cells, a move from senescence to proliferation was associated with decreased CD47.^48^ Thus, in primary noncancer and cancer cells, CD47 restrains growth. Yet, CD47 behaves in a dichotomous manner in its regulation of autophagy in primary^49^, ^50^ versus cancer cells.^51^

**FIGURE 2:**
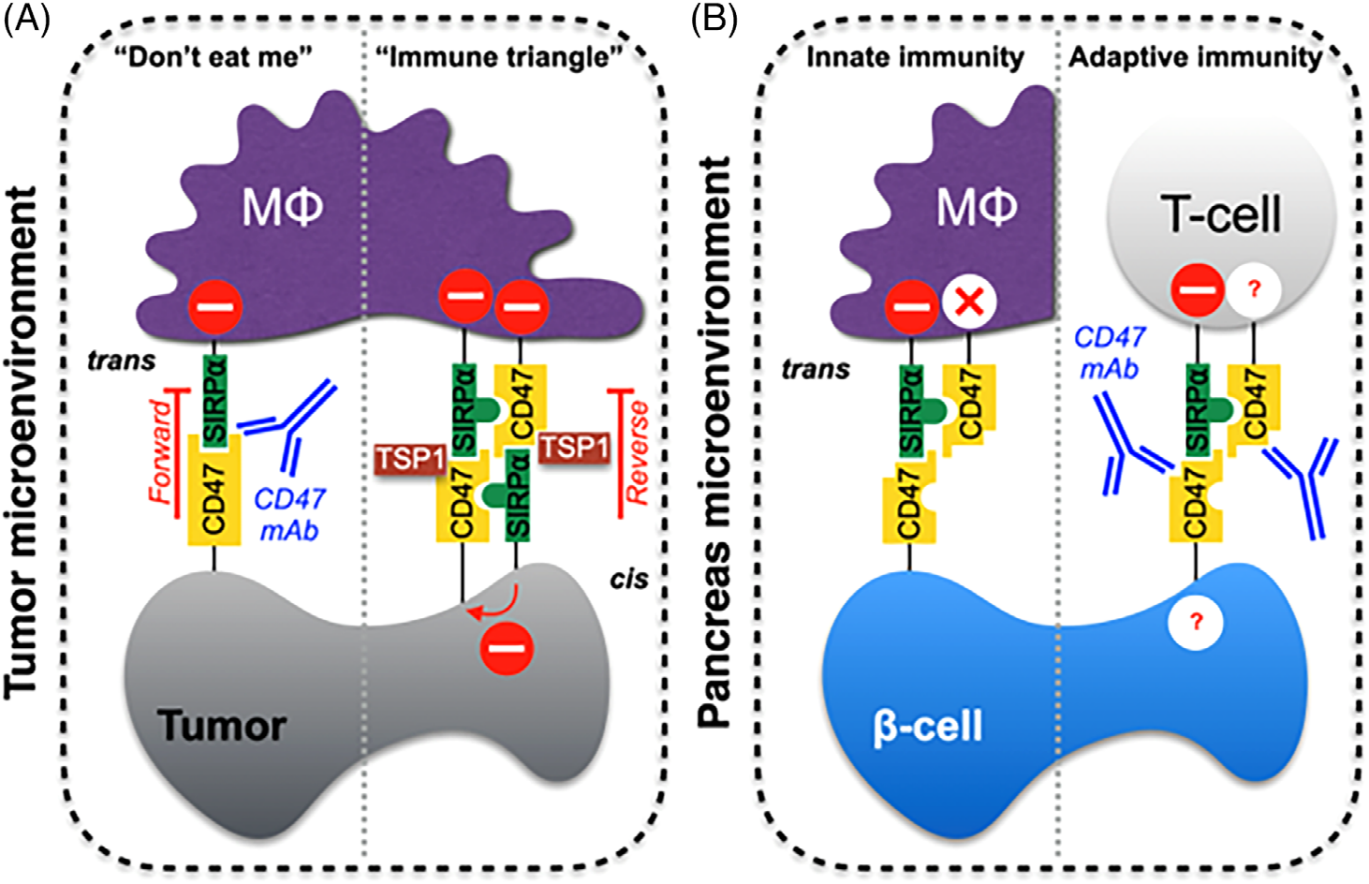
TSP1‐CD47‐SIRPα interactome in cancer immunotherapy and the case for type 1 diabetes. The conventional ‘don't eat me signal’ mediated by the forward negative effect in *trans* of CD47 on macrophages, postulated to be interfered with current blocking antibodies (mAbs). An inclusive ‘Immune triangle’ views the TSP1‐CD47‐SIRPα interactome converging multiple checkpoints, with the SIRPα central immune regulatory role also acting as a multitier protection both in *cis* and by reverse effect in *trans*, representing a natural mechanism of homeostasis to maintain peripheral tolerance (A). Absence of SIRPα in human β cells may increase vulnerability to self‐reactivity in genetically predisposed individuals by disrupting innate and adaptive immunity (B). SIRPα inhibitory effect in *cis*, as well as its reverse effect in *trans*, is potentially impaired. An on‐target effect of current CD47 immune checkpoint inhibitor mAbs damaging human β‐cell function and impacting T‐cell health should be entertained and elucidated. – indicates inhibitory effect; x inactive due to lack of SIRPα effect in *trans* and ? indicates unknown effect.

Similarly, CD47 perturbed cellular and organismal metabolism. Mutant mice lacking functional SIRPα had less plasma insulin and glucose tolerance.^52^ The role of CD47 in the disglycaemia was presumed but not shown. As fresh human islets from individuals without diabetes lacked SIRPα^53^ (Figure 2B), the relevance of the finding is unclear. Further miring things, human‐derived beta‐like EndoC‐βH1 cells, generated by multiple viral transfections, were employed to assess SIRPα.^54^ This distinction should be recalled when using beta‐like cell lines that are anticipated to show SIRPα expression^55^ in difference to human islets. Mice rendered hyperglycaemic by streptozotocin (STZ) showed less islet CD47 coincident with immune injury,^56^ although details on islet cell‐specific cell surface CD47 copy number (Bmax) were not obtained. Pertaining to global metabolic effects, young CD47‐null mice on a standard murine diet weighed less, exercised longer to exhaustion, and, at the cellular level, generated less pathologic ROS.^22^ Similarly, CD47‐null mice on a high fact diet (HFD) where lighter, had fewer adipocyte‐associated immune cells, and had preferable glucose tolerance.^57^ Several of these metabolic advantages, including better glucose balance, persisted in aged CD47‐null mice.^46^ However, extended intake of a HFD led to accentuated fatty liver and inflammation in null mice.^58^ In cell‐autonomous studies, CD47‐null islets showed superior function in vitro and where less sensitive to injury from STZ in vivo.^59^ This could be expected as removal of, or interference with, CD47 protected cells and animals from severe cytotoxic stress. Mice lacking CD47 showed cell and tissue protection from high‐dose regional^60^, ^61^ and lethal whole‐body radiation.^62^ In the absence of CD47, protection of bone marrow and immune cells^60^ and upregulation of helpful metabolic pathways contributed to improved outcomes.^63^ As well, a CD47 blocking antibody limited chemotherapy killing of cardiac myocytes, attendant fibrosis, and heart dysfunction.^64^ As an aside, phosphorylation of SIRPα was recognised to be temperature sensitive,^65^ which is of interest since CD47 negatively impacted murine responses to cold^36^ and reduced levels of brown fat.^66^ What is more, SIRPα activation, a process mediated by CD47, antagonised growth hormone signalling.^67^ These data layout a pattern of negative activities, many involving TSP1, that CD47 facilitates to upset cellular and organismal homeostasis and metabolism.

### 1.2 CD47 balances immune cells

Sometimes, activated CD47 is not overtly noxious. Immune cell migration was greater in the presence of CD47^68^, ^69^ or when antibody‐activated^70^ probably secondary to influences on integrins. Mutant SIRPα mice showed less immune cell migration^71^ although the status of CD47 and integrins was not determined. This finding suggests that SIRPα, alone, or in crosstalk with CD47, modifies integrin activation.^72^ This is not so unusual as SIRPα phosphorylates Src homology 2 domain phosphatase‐1 (SHP1) and the latter regulates integrin signalling.^73^ So too, the SHP1 and −2 tyrosine phosphatases are effectors of signalling pathways^74^ that intersect immune cells^75^ and that do not involve SIRPα. Immune cells from CD47‐nul mice showed loss of CD8+ T‐cell‐stimulated effector function and reduced natural killer (NK) cell control of viral infection,^76^, ^77^ inferring that CD47 adds to immune cell capacity. This is mirrored in human T cells mutated to lack CD47.^78^, ^79^ In keeping with this, CD47‐null mice have decreased host defences.^69^, ^80^ These findings beg the question if clinical CD47 antibodies unfavourably adjust immune cell adhesion, migration and function (Figure 2A). NK cells without SIRPα resisted the suppressive action of overexpressed cancer cell CD47.^81^ However, in this case, use of the murine CD47 blocking antibody clone 301 complicates data interpretation as clone 301 was cell and tissue protective under stress and inflammation.^61^, ^82^, ^83^, ^84^ In contrast, TSP1‐CD47 signalling enhanced cytoskeletal reorganisation and augmented human macrophage macropinocytosis of low‐density lipoprotein.^85^ This finding warrants additional study as macropinocytosis permits antigen uptake by dendritic cells and secondary T‐cell activation.^86^, ^87^ One might suppose that CD47 antibodies will warp antigen processing and presentation. The finding of TSP1‐mediated macropinocytosis should also be inspected in relation to trogocytosis^27^ as both appear to be invoked by activated CD47. These data push back on the idea that SIRPα, on finding ‘less’ nonimmune CD47, leads to cell envelopment.^88^

### 1.3 CD47 signalling is nuanced and complicated by other partnerships

CD47 antibodies, through disruption of the CD47‐SIRPα interaction, and in concert with other signals like pro‐phagocytosis calreticulin,^89^, ^90^ stimulated cell consumption.^91^ Yet surprisingly, the same CD47‐SIRPα affiliation restricted phagocytosis of cells independent of calreticulin.^92^ This inconsistency is compounded by the lack of an autoimmune inflammatory phenotype in CD47‐null mice. Such mice lack cell surface CD47 but display immune cell surface SIRPα and yet are self‐tolerant. This is also the case for heterozygote CD47+/– mice. In mice, foetal and maternal blood mix during pregnancy^93^, ^94^ something that, in breeding null or heterozygote CD47 mice, should, but does not, touch off autoimmune reactions. If CD47 is a self‐recognition signal how can the null and heterozygote mice tolerate themselves (Figure 2). Adding to this conundrum is the finding that cells take up TSP1 via calreticulin LDL‐receptor‐related protein^95^ which itself stimulates phagocytosis.^96^ Importantly and contrariwise, exogenous TSP1, acting via CD47, lowered immune cell activities in general.^97^, ^98^, ^99^, ^100^, ^101^ For example, TSP1, through CD47, inhibited human T‐cell receptor signalling.^78^ Given that (i) TSP1 binds CD47 strongly to exclude SIRPα binding to CD47^102^ and that (ii) TSP1 is heightened with acute and chronic inflammation, it is possible that what was interpreted as CD47‐SIRPα effects on innate and acquired immunity was, partly or wholly, a TSP1‐CD47 driven effect. Clinical CD47 blocking antibodies could not parse out TSP1 versus SIRPα consequences since, as far as can be discerned, they have not been tested against TSP1 binding to CD47 and TSP1 binding to SIRPα.^33^ Regarding the latter, the signature domain of TSP1 that bound CD47 did not bind to SIRPα and a CD47 blocking antibody did not make a difference in TSP1‐mediated SIRPα activation.^33^

### 1.4 CD47 may be less ‘self‐ish’ than first thought

Using red blood cells from CD47‐null mice led to the notion that CD47 provided a marker of self.^88^ In these studies, young C57Bl6 mice were given red blood cells from same‐age C57Bl6 CD47‐null mice. The red blood cells that lacked cell surface CD47 had a shorter circulation half‐life. It was accepted that membrane CD47 delayed phagocytosis of the transplanted red blood cells. On the other hand, macrophages from mice lacking SIRPα phagocytised neural debris.^103^ Thus, lack of CD47 on target cells or lack of SIRPα on macrophages amplified phagocytosis (Table 1). In contrast, mice lacking SIRPα or CD47 retained CD47‐positive and CD47‐null red blood cells.^92^ Plus, TSP1 via CD47 controlled removal of red blood cells from the circulation.^104^ In these situations, it would be interesting to distinguish between age and sex as protein expression and binding affinity no doubt fluctuate consequent to these.

**TABLE 1:** Examples of divergent effects mediated by CD47 and SIRPα.

Species	Cells, tissue, organismal	Effect	Where some dichotomies occur	Possible explanation and references
**CD47 promotes and limits self‐renewal**				
*Homo sapiens*	Cancer cells	TSP1‐CD47 promoted tumour cell growth in vitro and in vivo	Activated CD47 promoted cancer self‐renewal	Activated CD47 does not intersect/suppress OSKM in cancers^21^
*Homo sapiens Mus musculus*	Immune and nonimmune cells	TSP1‐CD47 suppressed cell growth	Activated CD47 decreased self‐renewal	Activated CD47 limits self‐renewal factors Oct3/4, Sox2, Klf4, and cMyc (OSKM)^42^, ^43^, ^44^, ^62^
**CD47 controls autophagy to protect or injure cells**				
*Homo sapiens Mus musculus*	Cancer cells	CD47 did not alter autophagy	CD47 blockade did not alter autophagy activity	CD47 is not a cell‐autonomous signal in cancers^51^
*Homo sapiens Mus musculus*	Immune and nonimmune cells	CD47 suppressed stress‐induced autophagy	CD47 blockade increased autophagy under stress to protect cells	CD47 is upstream of central autophagy pathways^49^, ^50^
**CD47 or SIRPα impacts positively and negatively immune cells and xenotransplantation**				
*Homo sapiens Sus scrofa*	Heart	Xenotransplantation	Porcine heart carrying human CD47 was rejected, although donor viral pathogens may have promoted organ loss	CD47 may not be a sufficient cloak for xenotransplantation^162^, ^182^
*Homo sapiens*	Stem cells	Xenotransplantation	Stem cells with extra cell surface CD47 were killed when transplanted into mice	CD47 is not a cloak to immune attack^153^; CD47 does not bind with correct affinity to mouse SIRPα^142^
*Homo sapiens*	Stem cell‐derived endothelial cells	Immune cell injury	Target cell display of a SIRPα interacting molecule limited immune injury	SIRPα may signal in a ‘reverse’ *trans* manner; SIRPα can act as a ligand for CD47 to suppress immune activity^167^
*Homo sapiens*	Neutrophils	Trogocytosis of T cells	A CD47 Ab that does not block SIRPα binding increased cell membrane consumption	CD47 promotes cell internalisation separate from SIRPα^28^
*Homo sapiens*	T cells	Human CD47 blocking Ab clone B6H12 decreased T cell cell‐autonomous activities	*Trans* CD47‐SIRPα signalling is not needed to decrease immunogenicity	CD47 Abs are able to decrease T‐cell activity irrespective of *trans* CD47‐SIRPα disruption; Ab effects on *cis* CD47‐SIRPα could not be excluded^169^
*Homo sapiens*	Macrophages	Phagocytosis of debris/antigens	TSP1‐CD47, separate from SIRPα, increased macrophage uptake of debris	CD47 Ab may alter natural ligand effects on engulfment^85^
**Phagocytosis is driven by TSP1 and may or may not be driven by CD47‐SIRPα**				
*Homo sapiens*	Macrophages	Chemotherapy increased rhabdomyosarcoma phagocytosis	Interruption of CD47‐SIRPα with CD47 blocking Ab did not increase phagocytosis	Human CD47 blockade does not always increase phagocytosis of cancer^183^
*Homo sapiens*	Macrophages	Calreticulin and interruption of CD47‐SIRPα increased phagocytosis	Interruption of CD47‐SIRPα alone did not increase phagocytosis	Secondary proinflammatory signals are required to drive SIRPα‐ stimulated phagocytosis^96^
*Homo sapiens*	Immune cells and fibroblasts	Secreted TSP1 binds calreticulin for removal from cells	Calreticulin promotes phagocytosis	TSP1 via calreticulin is an engulfment signal independent of CD47‐SIRPα^90^, ^92^
**Red blood cell clearance is regulated by more than CD47**				
*Homo sapiens*	Red blood cells	TSP1 Ab or CD47 Ab altered red cell membrane stiffness and induced apoptosis	TSP1 Ab or CD47 Ab made red blood cells phagocytotic targets in a cell‐autonomous manner	CD47‐SIRPα is not the dominant ‘don't eat me’ signal^116^
*Homo sapiens*	Red blood cells	Human red blood cells only bound to SIRPα at supraphysiologic Bmax	CD47‐SIRPα was presumed to be a major check on phagocytosis	Alteration or loss in CD47‐SIRPα does not drive human red blood cell clearance^119^
*Homo sapiens*	Red blood cells	CD47 did not restrain but increased phagocytosis of aged human red blood cells	CD47 was putatively a break on phagocytosis	CD47 promotes red cell phagocytosis; CD47‐SIRPα is not the dominant ‘don't eat me’ signal^117^
*Homo sapiens*	Red blood cells	Autoimmune anaemia	CD47 on red blood cells (Bmax) was the same between anaemic individuals and nonanaemic controls	Cell surface CD47 Bmax did not impact red cell autoimmune removal^131^
**Human islet endocrine cells lack cis CD47‐SIRPα signalling**				
*Homo sapiens*	Islet beta and endocrine cells	Absence of SIRPα at baseline and after cytokine stress	Islet endocrine cells are SIRPα negative	SIRPα is not a signalling molecule for human islet endocrine cells;^53^ SIRPα loss is human islet protective
*Homo sapiens*	Stem cell‐derived viral transfected beta‐like cells	Transfected cells showed SIRPα that responded to interleukin stimulation	Immortalised cell lines expressed SIRPα	Mutant endocrine cell SIRPα expression may not mirror primary human islet endocrine cell expression^55^
**CD47 blocking antibodies protect in situations with increased immune cell activity**				
*Mus musculus*	Immune cells	CD47 Ab clone 301	CD47 blockade decreased cell, tissue, and organ injury	CD47 blockade was associated with suppressed immune cell activity^82^, ^84^
*Mus musculus*	Natural killer cells	CD47 Ab clone 301 interrupted CD47‐SIRPα binding	CD47 Ab clone 301 increased immune cell killing	CD47 Ab blockade increased immune cell activation and injury^81^
**Mutant cells and mice lacking CD47 are not subject to autoimmune injury**				
*Mus musculus*	Cells, organismal	CD47‐SIRPα restrained phagocytosis	CD47‐null immune cells, that display SIRPα, did not attack nonimmune CD47‐null cells	Loss of immune SIRPα does not activate phagocytosis^126^
*Mus musculus*	Macrophages, red blood cells	CD47‐SIRPα limited phagocytosis	CD47‐ or SIRPα‐null mice did not phagocytise CD47‐null red blood cells	CD47 is not a self‐recognition signal^92^
**Bone marrow, tissue, and organ transplantation are improved with CD47 blockade**				
*Mus musculus*	Bone marrow, organs	Transplant acceptance	CD47‐null bone marrow and organs are accepted by CD47‐SIRPα positive animals	CD47 itself is not a major self‐recognition signal^83^, ^130^
*Mus musculus*	Syngeneic islet transplantation	CD47 Ab clone 301 improved islet engraftment and survival	CD47 Ab clone 301 disrupted CD47‐SIRPα	Loss of CD47‐SIRPα does not lessen syngeneic islet engraftment^59^
*Mus musculus Sus scrofa*	Organs	Transplant acceptance improved with CD47 blocking Ab	Mice given CD47 blocking Ab had better syngeneic and allo organ engraftment	Blockade of CD47‐SIRPα improves organ transplantation^134^, ^164^, ^165^
*Mus musculus*	Organs	Transplant acceptance improved by lowering but not blocking CD47	Mice given an agent that lowered CD47 protein levels showed improved transplant engraftment	Less CD47 does not incite immune injury and transplant loss^83^
*Mus musculus*	Organismal	CD47‐SIRPα limited autoimmunity	SIRPα‐null mice did not show autoimmunity	SIRPα alone is not a major restraint on inflammation
*Mus musculus*	Organismal	CD47‐SIRPα limited autoimmunity	CD47‐null homo and heterozygous mice did not show autoimmunity	CD47 alone is not a major restraint on inflammation
**CD47 blockade and cancer confirmation studies gave different results**				
*Mus musculus*	Immune cells	Immune cell attack and phagocytosis of orthotopic murine breast cancer	CD47 blocking Ab decreased tumour growth and increased immune cell activity towards tumours	CD47 Ab blockade increases immune attack of cancer cells
*Mus musculus*	Immune cells	Immune attack of and phagocytosis of orthotopic murine breast cancer	CD47 blocking Ab did not decrease tumour growth or immune cell activity towards tumours	CD47‐SIRPα Ab blockade did not increase immune attack of some tumours; some tumours regressed spontaneously^122^
**CD47 cointeractions with other membrane receptors**				
*Homo sapiens*	Endothelial cells	Blood flow and angiogenesis	CD47‐VEGFR coassociated and support VEGF signalling	TSP1‐CD47 decreased VEGF signalling^1^
*Bos taurus Sus scrofa*	Endothelial cells, vascular smooth muscle cells	Vascular biogas nitric oxide and cyclic nucleotide signalling	CD36 and CD47 suppressed vascular cell signalling	Separate from SIRPα, activated CD47‐CD36 limited proangiogenic nitric oxide and cGMP^181^
*Homo sapiens*	T cells	Cell survival	CD47 coassociated with FAS	CD47 was necessary for FAS‐mediated cell death^2^
*Homo sapiens*	Erythroleukaemia cells	Cell adhesion	CD47 coassociated with beta integrins	Cell adhesion through CD47‐integin^3^

Reflection on the role of biomechanical forces in phagocytosis could be advantageous in understanding the ‘self‐ish’ side of CD47. A yeast two‐hybrid analysis located PLIC1 and 2 that interacted with the cytoplasmic tail of CD47 and with cytoskeletal vimentin intermediate filaments.^105^ And as pointed out, CD47 has dealings with integrins,^106^ although integrins may not be needed for CD47‐SIRPα signalling.^107^ Notwithstanding, these bridging relationships could inform mechanistic insights germane to CD47‐mediated effects on phagocytosis. Indeed, monocytes from individuals with defective intermediate filaments had impaired phagocytosis.^108^, ^109^ As well, T regulatory^110^ and dendritic cells^111^ were controlled, in part, by vimentin intermediate filaments implicating in immune settings a possible CD47 regulation of cytoskeleton proteins. These data encourage a closer look at other cytoskeletal proteins such as myosin with CD47^112^ and actin with CD47 and TSP1.^113^ In human cells, TSP1 signalled through the actin cytoskeleton^114^ putatively via integrins or PLICs. In vitro, CD47‐SIRPα restrained phagocytosis but this was overwhelmed by target cell membrane stiffness^112^ implying that nonimmune cell cytoskeletal and membrane biomechanical properties dominated phagocytosis. This was demonstrated using red blood cells with variant membrane rigidity. Given that CD47, via the Rhesus factor (Rh) complex, interacts with red cell cytoskeletal proteins such as ankyrin,^115^ the clearance of murine CD47‐null red blood cells after transfusion into CD47‐positive animals^69^ may reflect the importance of target cell membrane stiffness rather than a unique ‘self‐ish’ feature of CD47. Most convincingly, red blood cells incubated with soluble TSP1 or a CD47 blocking antibody, each alone sufficient to incite phagocytosis, underwent alterations in membrane stiffness and subsequent cell death^116^ which would guarantee cell clearance. In vitro and in vivo experiments of CD47‐SIRPα phagocytosis are lacking in consideration of TSP1, the natural omnipresent ligand of CD47. And murine studies did not take into account this or age. When the scenario was reversed, enforced CD47‐SIRPα signalling actually enhanced phagocytosis of aged red blood cells.^117^ Thus, depending on age, the CD47‐SIRPα signal either restrained or improved phagocytosis. Of import, TSP1 and peptide 4N1K also increased phagocytosis of aged red blood cells.^117^ This is interesting, as TSP1 and CD47 were overexpressed in aged animals and in tissues from older people.^46^ Coming at things from another direction, human cancer cells treated with a SIRPα blocking antibody showed cytoskeletal changes.^118^ As prelude to phagocytosis, CD47‐dependent red blood cell adhesion to SIRPα only occurred at a SIRPα concentrations well above levels naturally found on immune cells.^119^ In studies that jump‐started interest in CD47 targeting, blocking antibody B6H12 enlarged macrophage activity against cancer cells while subsequently stimulating T cells.^120^ However, it is disquieting that attempts at confirming the results proved unsuccessful.^121^, ^122^ One wonders if in aged cells any prophagocytic posture of CD47‐SIRPα is a manifestation of revised binding affinities between soluble TSP1 and membrane bound CD47 or SIRPα. It is tantalising to think that cells wishing to avoid pruning might adjust the ‘age’ (binding affinity) of the CD47 or SIRPα ectodomains or both rather than altering Bmax or aggregation on the membrane. Elsewhere, CD47 was located proximate to and promoted aging. Human organs from individuals that were healthy showed more CD47 expression as a function of increased age contemporary with decreased self‐renewal transcription factors.^46^ Meanwhile, aged CD47‐null mice had better‐quality thermoregulation of blood flow^47^ and a blocking CD47 antibody improved ischemic tissue blood flow in aged mice^45^ and in vitro angiogenic activity in aged human arteries.^46^

In another example of ‘non‐self’ CD47, TSP1 and an antibody binding to CD47 increased membrane expression of phosphatidylserine, a robust prophagocytosis signal.^123^ Subverting the position of ‘self‐ish’ CD47, macrophage‐expressed CD14 controlled phagocytosis of apoptotic cells.^124^ CD47 associated with CD14 in lipid rafts but, when cells were challenged with a prophagocytic signal, CD47 disassociated.^6^ In turn, TSP1 promoted CD14‐mediated tolerance in macrophages.^125^ The many points already laid out and those to follow call for another look at the relationship between CD47 and self‐tolerance (Table 1).

### 1.5 CD47‐null is accepted by and lives in the SIRPα‐positive world

Bone marrow cells from CD47‐positive mice were given to irradiated CD47‐null mice where they engrafted.^126^ Later, macrophages from the chimeric mice, that carried cell surface SIRPα, did not phagocytise CD47‐null spleenocytes. If lack of target cell CD47 is the trigger for phagocytosis, then SIRPα‐positive macrophages should not tolerate CD47‐null spleenocytes. This is in line with findings that CD47‐positive bone marrow engrafted less fully in CD47‐positive recipients than CD47‐null marrow in CD47‐null recipients.^127^ But, in some cell transfer studies, the data are mixed. Specifically, CD47‐positive immune cells both suppressed^128^ or stimulated^129^ inflammation.

Shifting to complex structures, tissues and organs from CD47‐null animals survived and prospered when transplanted into CD47‐positive animals, which, parenthetically, housed SIRPα‐positive immune cells. In fact, CD47‐null full thickness skin grafts^83^ and CD47‐null hearts^130^ fared better after transplantation into CD47‐positive animals versus CD47‐positive organs. This should not transpire since the lack of nonimmune cell surface CD47 is expected to release the inhibitory signals mediated via immune cell SIRPα. Of translational bearing, cell surface levels of CD47 were the same between individuals with immune haemolytic anaemia and nonanaemic healthy individuals^131^ implying that immune cell attack of red blood cells was not influenced by variation in levels of cell membrane CD47. And an individual with a hereditary mutation in red blood cell membrane protein 4.2, which resulted in a complete loss of membrane CD47 protein showed only a mild drop in haemoglobin and a normal red blood cell count.^132^ Specific information on immune cell function in these individuals was not provided. Related to this are results from organ transplantation where CD47 targeting molecules were given. Mice^43^, ^133^ treated with a CD47 antibody (IgG2a clone 301), that blocked CD47‐SIRPα and strengthened phagocytosis,^134^ showed greater organ engraftment and function after allogeneic transplantation. In mice, this same antibody (clone 301) enhanced pancreatic islet outcomes after transplantation.^59^ Similar successes were reported in solid organ transplantation in rats^135^, ^136^ again using a CD47 blocking antibody (clone OX101) that interfered with CD47 binding to SIRPα and bettered phagocytosis.^137^ Thus, CD47 antibodies that undid the checkpoint ‘don't eat me’ signal provided by CD47‐SIRPα actually improved transplantation outcomes. This too pressures re‐evaluation of the CD47 ‘self‐ish’ postulate.

Also unanswered is the puzzle of the *cis* CD47‐SIRPα signal resident on immune and many nonimmune cells.^138^ The *cis* signal is a constitutive inhibitor of human immune and nonimmune cell activation and inflammation.^139^ Thus, even if CD47 expression was decreased or absent on target nonimmune cells to foster situational loss of *trans* signalling, immune cell *cis* CD47‐SIRPα should wholly or partly restrain activation. The same applies to the effects of CD47 blocking antibodies that presumably target just the *trans* CD47‐SIRPα signal (Figure 2). At any rate, whether clinical and pre‐clinical CD47 blocking antibodies are indiscriminate and simultaneously exert influence on *cis* and *trans* CD47 interaction with SIRPα has yet to be ascertained. This is not minor as the biologic impacts could yield negative repercussions. In some cells, engagement of the *trans* CD47‐SIRPα signal resulted in endocytosis of the complex^140^ and seemingly the anti‐inflammatory signal it mediated. Variance in the ratio of CD47 to SIRPα ectodomains was invoked to account for some of the inconsistencies in the CD47‐SIRPα story.^141^ Of note, a series of thoughtful experiments identified an optimal level of cell surface CD47 for maximal effect.^141^ Following this to its logical end, complete disruption of the CD47‐SIRPα connection, as with current CD47 blocking antibodies, hindered maximum phagocytosis. This is in keeping with bone marrow to kidney transplant studies, which denoted that either too much or too little CD47‐SIRPα binding resulted in graft rejection.^142^

### 1.6 CD47 protein‐targeting molecules are potential immunogens

Proteins including those employed as therapies^143^, ^144^ are primary activation molecules for human immune cells.^145^ The response to protein therapies is broad and includes innate and adaptive features.^146^, ^147^ The result of this can be less therapeutic activity^148^, ^149^ and for the immunogenic effect to dysregulate or inactivate the natural protein target. To wit, CD47 antibodies could be immunogenic. Moreover, antibodies formed to clinical CD47 antibodies might themselves be capable of signalling via CD47 or SIRPα. This process could propagate with each new antibody a source of immunogenicity. Available data do not include particulars on development of circulating antibodies to CD47 or SIRPα blocking molecules.^91^ Also not known is whether therapeutic CD47 antibodies are taken up by phagocytic cells for processing. But given the high affinity that CD47 clinical antibodies have for natural CD47,^91^ antibodies would likely be included bound to phagocytised target cells. Besides, as the CD47 ectodomain on immune cells interacts with CD47 antibodies, the complex may be cleared through routine cell membrane processing of the natural receptor.^150^ Screening for reactions to CD47 antibodies, circulating soluble CD47, TSP1, and SIRPα protein, and protein‐displaying exosomes,^151^, ^152^ by pointing out individuals that develop such responses, could allow refined use of the therapeutic agents. In turn, it might yield a portfolio of natural proteins that interact with human CD47 and change interactions with TSP1, SIRPα and other molecules.

### 1.7 More CD47 may or may not be better

CD47 is viewed as a protective cloak for cells,^153^, ^154^ organs^155^, ^156^, ^157^ and inanimate surfaces.^90^, ^158^, ^159^ Islet‐like cells altered to display exaggerated levels of CD47 transplanted into primates and diabetic humanised mice persisted and normalised blood glucose levels in the mice.^154^, ^160^ However, the long‐term persistence of the artificial islets, the status of overexpressed and natural CD47 and effects on downstream signalling were not mentioned. The cell‐autonomous repercussions of hyper‐CD47 expression have not been determined. But data suggest rapid shifts in CD47 may be deleterious, in part, by shutting down self‐renewal pathways and cMyc,^44^ a governor of much that cells do. And when mice received a clinical CD47 blocking antibody they were rendered hyperglycaemic, presumably from death of the CD47 overexpressing transplanted islets.^160^ Porcine cells and organs were conjectured to benefit from an extra display of human CD47^161^ while human CD47 bound porcine SIRPα.^28^ However, porcine monocytes phagocytised human red blood cells only modestly.^28^ Irrespective, biotechnology concerns are working to develop pigs that overexpress human CD47. A porcine heart with surplus human CD47 was given to an individual who did not qualify for allotransplantation and functioned for almost 7 weeks.^162^ Species‐specific viral pathogens could present a barrier to the process.^163^ However, reasons why overabundance is preferable over natural amounts of membrane CD47 are wanting. Answering this is especially important as a lack of CD47 and CD47 blocking antibodies were consistently protective in syngeneic^43^, ^136^, ^164^, ^165^ and allogeneic^130^ organ transplantation in small and large mammals. And disruption of CD47 signalling with blocking antibodies consistently resulted in greater blood flow and limited ischemia reperfusion damage,^24^, ^45^, ^84^, ^127^, ^166^ the latter a significant source of organ impairment and rejection after transplantation. The CD47 blocking antibodies deployed in these studies disrupted checkpoint CD47‐SIRPα inviting consideration of how to rank the ‘don't eat me’ signal in the context of transplantation. Furthermore, an analysis of a panel of cell surface cloaking proteins noticed that decorating with supplemental CD47 did not enhance immune control.^153^ Another ripple in the story is that excess of a nonfunctional SIRPα ‘engager’ (binding molecule) on target cells spared them from phagocytosis.^167^ The presumption was that target cells with the nonfunctional SIRPα engager were somehow acting through native immune cell SIRPα to turn down phagocytosis. But this leaves open the nature of the ‘engager’ and its effects on same‐cell *cis* interactions with bonafided CD47 coassociations. Even so, use of nonfunctional SIRPα engaging to defend against immune cell aggression merits study. Scrutiny of CD47, the cytoskeleton, *cis* and *trans* interface with other cell surface and soluble molecules and intracellular signalling pathways after overdosing with ectodomains and whole protein or portions thereof may be worthwhile.

### 1.8 Tolerating CD47

A CD47 antibody (10 μg/mL) and a C‐terminus‐derived TSP1 peptide (50 μg/mL) limited differentiation of precursor to Th1 cells.^168^ The antibody and peptide concentrations used in this study were at or above levels sufficient to activate other CD47‐mediated signals, including cell death. Unfortunately, the CD47 antibody clone was not specified. This is important as CD47 antibodies act as agonists and as antagonists. In these studies,^168^ the stated cell culture conditions would increase TSP1 production and secretion to levels with signalling impact, but this too was not assessed. With prolonged exposure to a CD47 antibody (IgG1 clone B6H12), mononuclear cells, on appropriate stimulation, yielded weak T cells that were still sensitive to cytokine support.^169^ These data showed that an antibody that blocks CD47‐SIRPα at concentrations as low as 0.3 μg/mL^102^ limited T‐cell processes. However, it is not clear what this implies for nonactivated CD47. Underperformance of CD47‐null T cells would also indicate that CD47 may implicitly change cell behaviour. Adding another kink, an antibody to signal regulatory protein gamma (SIRPγ), assumably via CD47, suppressed human T cells in vitro, while in mice, the antibody delayed onset of graft‐versus‐host disease.^170^ Then again, the roles of *trans* and *cis* SIRPα were not excluded. Sorting out these pathways is frustratingly complex given the multiple known *cis* versus *trans* coactions CD47 constitutively maintains.^171^ Findings in genetically engineered mice add to the disparate data. Mice with mutations that promote diabetes and that lack CD47 developed anaemia sooner and to a more severe degree than CD47‐positive mice,^172^ a fact taken as showing that CD47 was tolerant. At issue here is that blood glucose levels were not determined. CD47 plays a role in glucose homeostasis,^59^ while glycosylation of red blood cells, as seen in diabetes, refashions membrane stiffness,^173^ perhaps triggering tendencies to phagocytosis.^174^ And diabetes and anaemia are closely linked.^175^ While as noted, in vitro studies found that membrane stiffness overran the inhibitory effects of CD47‐SIRPα on phagocytosis.^112^

### 1.9 Points to consider on CD47, a red herring or a meaningful target

An exhaustive coverage of CD47 was not herein intended. Rather, an appraisal of select cell, animal, and human studies suggests that for CD47, (i) the data are conflicted, (ii) there remains much to understand, and (iii) the information from studies in other species may or may not pertain to human beings. The findings also emphasise that there are multiple important signals beyond SIRPα that CD47 regulates. The present paradigm, especially regarding cancer, is that only the CD47‐SIRPα signal counts and limited to innate immunity. Interestingly, CD47‐SIRPα may trigger both a pro‐ and anti‐phagocytic signaling. In all of this, the TSP1‐CD47 interaction was left unconsidered and remains untested in relation to SIRPα and CD47 antibodies, and in terms of the natural molecules. Furthermore, the identification of possible disruption of these many signals by CD47‐targeted interventions is needed. Finally, we suggest that TSP1 is the likely source of immune signalling through CD47 in *trans* and that SIRPα may be contributing by regulating CD47 in *cis*. These and other points challenge the predominant view of CD47‐SIRPα.

It behoves researchers to accept the ambiguity in CD47, acknowledge its pleiotropism, and tolerate the current setbacks taking them as an inflection point for finding meaningful approaches to cure cancer, autoimmune, and other inflammatory diseases. Closer appreciation for, and attention to, the natural soluble and cell surface CD47 interacting molecules is a reasonable place to begin. This should be coupled with analysis of the binding strengths and time course of these interactions under in vitro and in vivo occasions. Tracking immune and nonimmune cell membrane stiffness in situations of less or surfeit CD47 and SIRPα expression may clarify discrepancies. It is tenable that CD47 is linked too tightly to distinct homeostatic pathways to permit safe targeting by molecules that widely block ectodomain binding. Configuring molecules to provide single pathway activity will potentially unlock the benefits of CD47. As another option, the partial lowering of CD47 protein with RNA interference technology protected cells and animals and improved healing^61^, ^82^, ^83^, ^176^ under various stressors. Additionally, a method for selecting molecules other than antibodies that alter CD47 binding to SIRPα was described^177^, ^178^ but what such agents do in cells and in vivo is not clear. Otherwise, tacking a step back to its primary high‐affinity ligand TSP1, that, like CD47, is upregulated in inflammation, transplantation, metabolic conditions, cancer and other diseases,^179^, ^180^ could be an answer.

## AUTHOR CONTRIBUTIONS

J.S.I. and E.M. conceived the manuscript and composed the figures. J.S.I. and E.M. wrote the manuscript and approved the final draft.

## CONFLICT OF INTEREST STATEMENT

J.S.I. is a consultant to San Rocco Therapeutics, Tampa, FL. E.M. declares no conflicts of interest regarding the work.

## AI STATEMENT

The authors confirm that artificial intelligence‐assisted technologies of any sort were not employed in the creation of the manuscript.
